# Bleeding Diathesis as the Initial Presentation of Chronic Myeloid Leukemia: A Case Series

**DOI:** 10.7759/cureus.37201

**Published:** 2023-04-06

**Authors:** Farjah H AlGahtani, Leena Alshaman, Ghada ElGohary, Aamer Aleem, Fatmah S AlQahtany

**Affiliations:** 1 Division of Hematology/Oncology, Department of Medicine, College of Medicine, King Saud University, King Saud University Medical City, Riyadh, SAU; 2 Hematology and Oncology, Division of Hematology/oncology, College of Medicine, King Saud Medical City King Saud University, Riyadh, Saudi Arabia, and Departement of Adult Hematology/internal Medicine, Faculty of Medicine, Ain Shams University, Cairo, Egypt, Cairo, EGY; 3 Hematopathology Unit, Department of Pathology, College of Medicine, King Saud University, King Saud University Medical City, Riyadh, SAU

**Keywords:** bcr-abl, tyrosine kinase inhibitor (tki), chronic phase, philadelphia chromosome, chronic myeloid leukemia

## Abstract

Chronic myelogenous leukemia, or CML, is another name for chronic myeloid leukemia (CML), a cancer type that starts in certain bone marrow blood-forming cells. The primary initiator of granulocytic proliferation in CML, a myeloproliferative malignancy, is the BCR-ABL1 fusion protein or Philadelphia chromosome. CML is classified into three stages: chronic, accelerated, and blast. It has been widely recognized that the likelihood of developing CML varies by gender, geography, and age. In the chronic phase of CML (CML-CP), bleeding is a rare sign since the thrombocyte and coagulation functions are still adequate. Uncertainties exist regarding the CML bleeding mechanism. We report four cases of CML-CP in adult patients. The majority of these patients had CML and had idiopathic spontaneous bleeding in multiple locations.

## Introduction

Chronic myeloid leukemia (CML), also known as chronic myelogenous leukemia, is a cancer type that starts in certain bone marrow blood-forming cells [1]. CML, which is a myeloproliferative tumor, results from granulocytic proliferation, which is mostly caused by the BCR-ABL1 fusion protein or the Philadelphia chromosome [2]. CML is classified into chronic, accelerated, and blast phases. Now that there are many effective treatments for CML in its chronic phase (CP), the accelerated and blast phases occur less frequently. The isoform p210 is present in 95% of patients with CML [3]. Only 1-2% of CML patients have the p190 isoform, while 5-7% of patients have both the p210 and p190 isoforms [4,5]. It was discovered that patients with only the p190 isoform have various chromosomal abnormalities and hematological characteristics [4,5]. Recently, p190 was linked to an early entry into the blast phase and a reduced response to therapy with tyrosine kinase inhibitors (TKIs) [6-8]. It is well known that the prevalence of CML varies by gender and geographic region and rises with age [9]. In various regions, it varies from 0.4 per 100,000 people to 1.75 per 100,000 people [10]. We present four adult CML cases in the chronic phase (CML-CP). The majority of these patients had CML and had spontaneous bleeding in various areas without a clear explanation.

## Case presentation

Case 1

A 37-year-old man presented to the emergency department with complaints of night sweats, general fatigue, early satiety, left abdominal discomfort, and a history of weight loss of 10 kg over the last two months. He had previously consulted a local hospital and had been informed that he had a high white blood cell (WBC) count of 100,000 per microliter and splenomegaly of 20 cm based on an ultrasound of the abdomen. He had then been advised to seek further evaluation at a tertiary hospital center.

**Figure 1:**
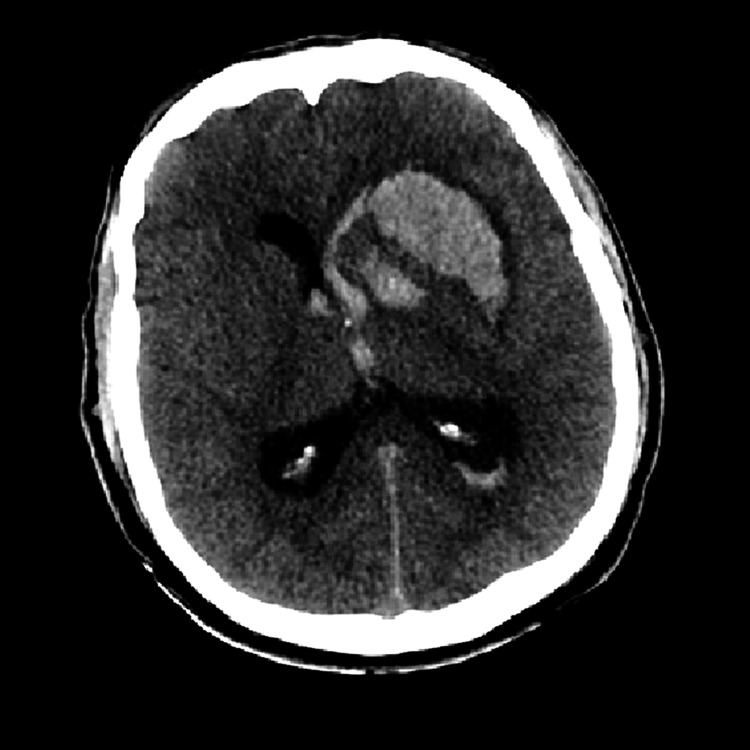
CT of the brain showing intraventricular hemorrhage CT: computed tomography

On further examination, the patient looked unwell, with abdominal distention and a palpable spleen measuring 14 cm below the costal margin. His lab results showed WBC of 434,300 per cubic millimeter (neutrophilia at 178.1 cells/µL, blast: 1%), RBC of 2.4 m/cmm, hemoglobin (Hb) of 74 g/L, hematocrit (HCT) of 22.90%, platelet (PLT) count of 251,000 per cubic millimeter, prothrombin time (PT) of 191 seconds, international normalized ratio (INR) of 1.49, partial thromboplastin time (PTT) of 42.80 seconds, and lactate dehydrogenase (LDH) of 1707 U/L. He was discharged on hydroxyurea (HU) 1 g twice a day (BID) and allopurinol. Two weeks later, he was brought to the emergency department with a decreased level of consciousness. He was also found to have a right-sided weakness and a history of vomiting six times before his presentation. On physical examination, his Glasgow Coma Scale (GCS) score was 8/15, responding only to painful stimuli. CT scan imaging revealed frontal lobe hemorrhage with a midline shift, intraventricular hemorrhage (IVH), and hydrocephalus (Figure 1). He was then admitted to the ICU and placed under sedation and on a mechanical ventilator. The neurosurgery team was consulted, and he was taken to the operating room (OR) for hematoma drainage and right frontal external ventricular drain (EVD) insertion. Cytoreduction therapy with cytarabine (one dose) and hydroxyurea (HU) 1 g three times a day (TID) was prescribed. Leukapheresis was not performed for this patient.

Following this, the lab tests showed WBC of 387,700 per cubic millimeter, RBC of 2.5 m/cmm, Hb of 73 g/L, PLT of 306,000 per cubic millimeter, PT of 19.90 seconds, INR of 1.57, PTT of 46.60 seconds (mixing study revealed weak lupus inhibitor), factor X 202%, factor vW activity of 160%, vWF assay of 197 IU/dL, and BCR-ABL ratio of 1.75 (normalized ratio: 175%). The patient was later extubated after undergoing a tracheostomy. His hospitalization was complicated by pneumonia, for which he was managed with broad-spectrum antibiotics. After he attained clinical improvement, he was discharged on dasatinib 70 mg once daily. He is currently doing fairly well; he is walking without assistance and has even returned to his daytime employment.

Case 2 

**Figure 2:**
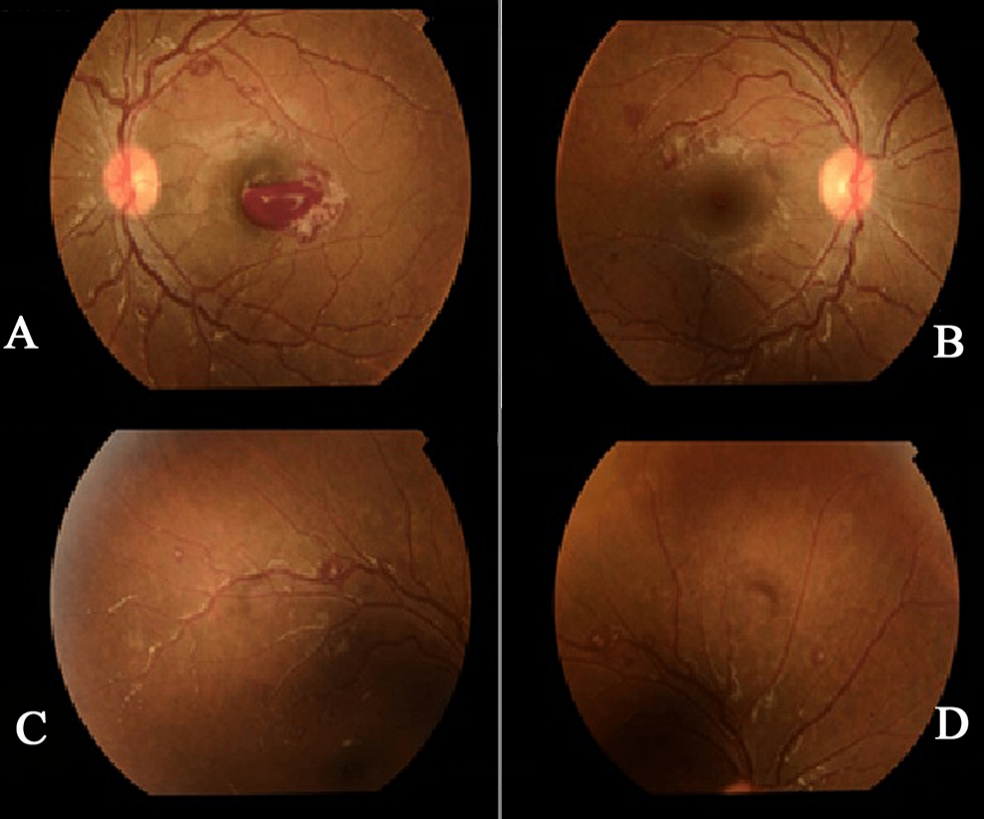
The fundus exam of the patient shows retinal hemorrhage and Roth's spots

A previously healthy 18-year-old woman presented to the ophthalmology emergency room with a complaint of blurry vision of a day's duration. The complete blood count (CBC) showed WBC of 225,000 per cubic millimeter (neutrophilia at 107.5 cells/µL), RBC of 2.9 m/cmm, Hb of 47 g/L, PLT of 255,000 per cubic millimeter, and LDH of 970 μl. A bone marrow examination and genetic studies were needed to establish a diagnosis. There were no coagulation profile parameters. The BCR-ABL ratio was 0.765, the normalized ratio was 76.5%, and the CT chest result was unremarkable. She was taken to the ophthalmology team. A fundus examination revealed multiple intraretinal hemorrhages with some white-centered hemorrhages (Roth’s spots) in the eyes, as shown in Figures 2A-2D. She was found to have leukocytosis and microcytic hypochromic anemia.

The BCR-ABL test showed positive results, and the platelet aggregation showed normal aggregation with all the used agonists. The peripheral blood smear (PBS) showed moderate leukocytosis with marked left-shifted granulocytes and basophilia (6%) and eosinophilia (7%), and some reactive lymphocytes were noted. The blast percentage was around 1-2% [11].

The hypercellular bone marrow for the patient’s age was consistent with the diagnosis of CML-CP morphologically and by molecular genetic testing. However, further correlation with the pending karyotype and fluorescence in situ hybridization (FISH) results was recommended to assess the disease phase.

Case 3

**Figure 3:**
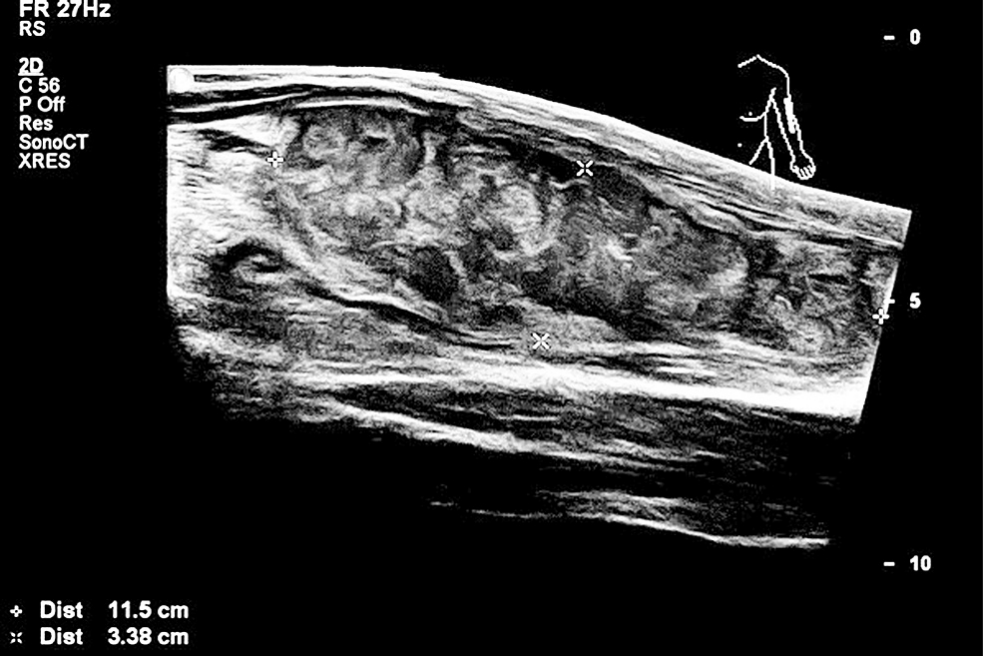
Ultrasonography of the elbow showing a hematoma

A 56-year-old male with known hypertension presented to the emergency department with an acutely painful erythematous discoloration that had erupted gradually on his left forearm over three days. It had extended to reach the area around his elbow. He had previously sought medical advice at a dermatology clinic, and lab investigations had shown a high WBC of around 450,000 per cubic millimeter; he had then been instructed to visit the emergency department for further evaluation. As shown in Figure 3, ultrasonography of the elbow showed significant erythematous discoloration that was petechial in nature and suggestive of compartment syndrome of the left forearm. There had been no previous similar events or a history of trauma, and there was no history of fever, but the patient did report experiencing night sweats and weight loss.

The peripheral blood findings showed hyperleukocytosis with marked neutrophilia and myelocyte bulge, as well as eosinophilia and basophilia. Blast cells accounted for 2% of the total. No Auer rods were seen. Platelets were mildly increased. Red cells were normochromic and monocytic. Nucleated red blood cells (NRBCs) were seen. The neutrophil alkaline phosphatase (NAP) score was 2 (normal score: 20-100).

The bone marrow examination revealed marked hypercellularity and left-shifted granulopoiesis. The findings were consistent with myeloproliferative neoplasms (MPN) and CML-CP. The laboratory results showed a WBC count of 418,700 per cubic millimeter, neutrophilia of 207.3, Hb of 75 g/L, RBC of 2.9 m/cmm, and PLT of 470,000 per cubic millimeter. The coagulation result showed PT of 17.10, INR of 1.32, PTT of 43.00 seconds, fibrinogen of 3.34 g/L, and D-dimer of 0.69. Platelet aggregation tests revealed a normal response to all agonists. He had an LDH of >1000 U/L, gamma-glutamyl transferase (GGT) of 117 u/l, and BCR-ABL/ABL ratio of 1.2182 (normalized ratio: 121.82%). The spleen was severely enlarged, measuring 28 cm, with a dilated splenic vein and no ascites.

Left-arm ultrasound findings showed minimal subcutaneous edematous changes. On color-flow Doppler analysis, there was a large intramuscular well-defined heterogeneous area in the medial aspect of the left forearm devoid of vascularity; it measured approximately 11.5 x 3.3 cm and raised suspicion for intramuscular hematoma for further evaluation. The Doppler ultrasound showed no signs of deep venous thrombosis (DVT).

The CT angiography of the upper extremity showed swelling and redness in the left forearm, which suggested a hematoma instead of a DVT. No venous thrombus or occlusion was seen. Good patency and caliber of the axillary artery, brachial artery, radial and ulnar arteries, as well as the interosseous artery, were observed. We confirmed the presence of a large, round, hypodense intramuscular collection in the flexor group of the left forearm muscles, measuring 4 x 6 x 11 cm, which was mildly heterogeneous and could represent a hematoma. It was decided that a follow-up ultrasound was required. Following the detection of chromosomal abnormality (T315I), lab sequencing of the BCR/ABL fusion gene revealed no mutation.

Case 4

A 52-year-old woman with diabetes mellitus and high blood pressure presented with bleeding in her retina. She had a high WBC and a positive BCR-ABL. After laser treatment, she was found to have a retinal hemorrhage and a WBC count of 160,000 per cubic millimeter. She denied having a history of chest pain, shortness of breath (SOB), syncope, neurological symptoms, headache, peripheral numbness, dizziness, abdominal pain, or a change in bowel habits. The B symptoms showed weight loss and a loss of appetite, as well as a skin rash and joint pain. The patient was admitted to the hospital as a case of likely CML based on leukocytosis of 151K and a 3% blast in the PBS. The eosinophil and basophil counts were high. Peripheral blood morphology showed marked leukocytosis, mainly in neutrophils and myelocytes, and all maturation sequences were seen. The blasts were around 2%, the basophils were 15%, and the granulocytes were 83%. There were normocytic, normochromic RBCs with mild anisopoikilocytosis, reflected by the presence of some ovalocytes, and elliptocytes. Nucleated RBCs were seen, and mild thrombocytosis and a few large forms were noted. All of the above findings were associated with myeloproliferative disorders and suggested CML. The BCR-ABL1 was positive, and the bone marrow showed signs consistent with CML. The patient's vitals were within normal range, with no signs of lymphadenopathy, skin rash, jaundice, or lower limb swelling.

## Discussion

Based on a population-based registry designed to identify the incidence of CML in 20 European countries, the incidence of CML was 0.39 per 100,000 people per year in people aged 20-29 years old and increased with age to 1.52 in people over 70 years old [12]. Furthermore, it was specified that the highest incidence was in Italy (1.39) and the lowest was in Poland (0.69) [12]. Additionally, investigators have studied the trend of CML incidence in the United States from 1975 to 2009 and found that the incidence was 1.75 per 100,000 people per year and increased with age [13]. The highest incidence was reported in Detroit, and the lowest was among people of Asian descent [13].

Patients with CML tend to bleed mostly due to platelet dysfunction, and bleeding diathesis is uncommon (<10%) in these patients [14]. Megakaryocytes that are dysfunctional are thought to cause a clonal increase in platelet dysfunction in CML. Therefore, a BCR-ABL-targeting therapy would be equally successful in reducing CML blasts and abnormal megakaryocytes [14]. Coagulation studies were closely monitored in the event of bleeding [15].

CML may first manifest as atypical bleeding that may be linked to a factor XIII (FXIII) deficiency. For CML patients who initially present with bleeding that cannot be distinguished by the standard clotting screening test, it is crucial to screen for coagulopathy, especially FXIII activity, and to supplement plasma [16]. The majority of cases are incidental findings on routine CBC and have B symptoms like fever, loss of weight, and night sweats [17]. Acute leukemia may initially manifest as bleeding; however, it is uncommon for CML patients, particularly in the chronic phase, to present with early spontaneous bleeding without coagulopathy and thrombocytopenia [15]. CML patients tend to bleed mostly due to platelet dysfunction, and bleeding diathesis is rare (<10%) in this patient population [17].

The early, chronic phase, which lasts for a median of four to six years in CML, is characterized by an excess of immature myeloid cells and mature granulocytes. For the treatment of CML, various TKIs have been developed. Imatinib mesylate, a first-generation BCR-ABL TKI, and dasatinib and nilotinib, second-generation TKIs, have significantly improved the prognosis of CML [18]. By blocking BCR-ABL kinase, TKIs have greatly improved the chances of favorable outcomes in CML. One of the most popular and first-line TKI treatments for CML is imatinib [19].

According to a case report, the fundus examination of a 19-year-old healthy woman who experienced a sudden, painless loss of vision in her left eye for one day also revealed the same result as in case 2: multiple intraretinal hemorrhages with some white-centered hemorrhages (Roth's spots) in four quadrants in both eyes [20]. In another reported case, the peripheral smear revealed the same result as in case 3: marked leukocytosis with significant neutrophilia and increased granulation in some cells [21].

## Conclusions

The mechanism of bleeding in CML is unknown. Several studies have suggested that bleeding may be caused by leukemic cell metastasis, clotting factor defects, interferon-induced clotting factor inhibition, or platelet dysfunction caused by a TKI. It is widely accepted that the likelihood of developing CML varies by gender, geographic location, and age. Due to platelet dysfunction, CML patients frequently bleed, and bleeding diathesis is unusual in these patients. We discussed four adult cases of CML-CP. Most of the patients presented with an incidental finding on a routine CBC and with B symptoms like fever, loss of weight, and night sweats. More case studies are needed to establish bleeding diathesis as an initial symptom of CML.
